# Better Existing Water, Sanitation, and Hygiene Can Reduce the Risk of Cholera in an Endemic Setting: Results From a Prospective Cohort Study From Kolkata, India

**DOI:** 10.1093/ofid/ofad535

**Published:** 2023-11-21

**Authors:** Md Taufiqul Islam, Justin Im, Faisal Ahmmed, Deok Ryun Kim, Birkneh Tilahun Tadesse, Sophie Kang, Farhana Khanam, Fahima Chowdhury, Tasnuva Ahmed, Md Golam Firoj, Asma Binte Aziz, Masuma Hoque, Juyeon Park, Hyon Jin Jeon, Suman Kanungo, Shanta Dutta, Khalequ Zaman, Ashraful Islam Khan, Florian Marks, Jerome H Kim, Firdausi Qadri, John D Clemens

**Affiliations:** International Centre for Diarrhoeal Disease Research, Bangladesh, Dhaka, Bangladesh; Epidemiology, Public Health, Impact Unit, International Vaccine Institute, Seoul, Republic of Korea; International Centre for Diarrhoeal Disease Research, Bangladesh, Dhaka, Bangladesh; Epidemiology, Public Health, Impact Unit, International Vaccine Institute, Seoul, Republic of Korea; Epidemiology, Public Health, Impact Unit, International Vaccine Institute, Seoul, Republic of Korea; Epidemiology, Public Health, Impact Unit, International Vaccine Institute, Seoul, Republic of Korea; International Centre for Diarrhoeal Disease Research, Bangladesh, Dhaka, Bangladesh; International Centre for Diarrhoeal Disease Research, Bangladesh, Dhaka, Bangladesh; International Centre for Diarrhoeal Disease Research, Bangladesh, Dhaka, Bangladesh; International Centre for Diarrhoeal Disease Research, Bangladesh, Dhaka, Bangladesh; Epidemiology, Public Health, Impact Unit, International Vaccine Institute, Seoul, Republic of Korea; International Centre for Diarrhoeal Disease Research, Bangladesh, Dhaka, Bangladesh; Epidemiology, Public Health, Impact Unit, International Vaccine Institute, Seoul, Republic of Korea; Epidemiology, Public Health, Impact Unit, International Vaccine Institute, Seoul, Republic of Korea; Cambridge Institute of Therapeutic Immunology and Infectious Disease, University of Cambridge School of Clinical Medicine, Cambridge Biomedical Campus, Cambridge, United Kingdom; National Institute of Cholera and Enteric Diseases, Indian Council of Medical Research, Kolkata, West Bengal, India; National Institute of Cholera and Enteric Diseases, Indian Council of Medical Research, Kolkata, West Bengal, India; International Centre for Diarrhoeal Disease Research, Bangladesh, Dhaka, Bangladesh; International Centre for Diarrhoeal Disease Research, Bangladesh, Dhaka, Bangladesh; Epidemiology, Public Health, Impact Unit, International Vaccine Institute, Seoul, Republic of Korea; Cambridge Institute of Therapeutic Immunology and Infectious Disease, University of Cambridge School of Clinical Medicine, Cambridge Biomedical Campus, Cambridge, United Kingdom; Madagascar Institute for Vaccine Research, University of Antananarivo, Antananarivo, Madagascar; Heidelberg Institute of Global Health, University of Heidelberg, Heidelberg, Germany; Epidemiology, Public Health, Impact Unit, International Vaccine Institute, Seoul, Republic of Korea; International Centre for Diarrhoeal Disease Research, Bangladesh, Dhaka, Bangladesh; International Centre for Diarrhoeal Disease Research, Bangladesh, Dhaka, Bangladesh; Epidemiology, Public Health, Impact Unit, International Vaccine Institute, Seoul, Republic of Korea; Department of Epidemiology, Fielding School of Public Health, University of California, Los Angeles, Los Angeles, California, USA

**Keywords:** cholera, endemic settings, WASH

## Abstract

**Background:**

Global cholera control efforts rely heavily on effective water, sanitation, and hygiene (WASH) interventions in cholera-endemic settings.

**Methods:**

Using data from a large, randomized controlled trial of oral cholera vaccine conducted in Kolkata, India, we evaluated whether natural variations in WASH in an urban slum setting were predictive of cholera risk. From the control population (n = 55 086), baseline WASH data from a randomly selected “training subpopulation” (n = 27 634) were analyzed with recursive partitioning to develop a dichotomous (“better” vs “not better”) composite household WASH variable from several WASH features collected at baseline, and this composite variable was then evaluated in a mutually exclusive “validation population” (n = 27 452). We then evaluated whether residents of better WASH households in the entire population (n = 55 086) experienced lower cholera risk using Cox regression models. Better WASH was defined by a combination of 4 dichotomized WASH characteristics including safe source of water for daily use, safe source of drinking water, private or shared flush toilet use, and always handwashing with soap after defecation.

**Results:**

Residence in better WASH households was associated with a 30% reduction in risk of cholera over a 5-year period (adjusted hazard ratio, 0.70 [95% confidence interval, .49–.99]; *P* = .048). We also found that the impact of better WASH households on reducing cholera risk was greatest in young children (0–4 years) and this effect progressively declined with age.

**Conclusions:**

The evidence suggests that modest improvements in WASH facilities and behaviors significantly modify cholera risk and may be an important component of cholera prevention and elimination strategies in endemic settings.

**Clinical Trials Registration.** NCT00289224.

## Teaser

Cholera control relies on effective water, sanitation, and hygiene (WASH) interventions in cholera-endemic settings. Modest improvements in WASH facilities and practices significantly modify the risk of cholera, which may be an important component of cholera prevention and elimination strategies.

## Graphical Abstract

**Graphical abstract:**
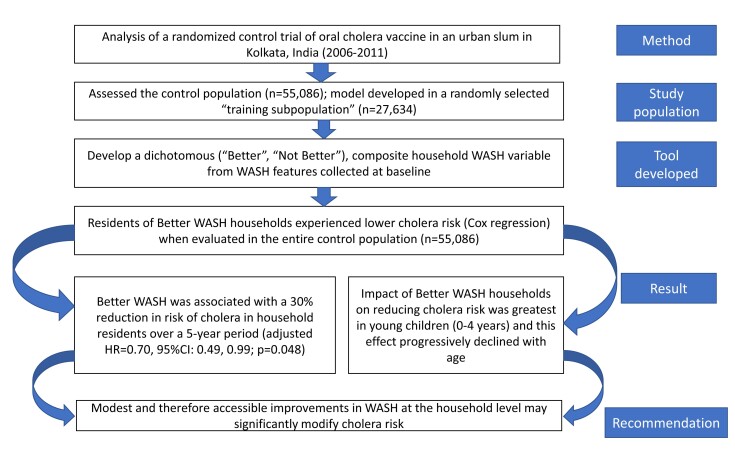

Cholera remains an important public health problem, particularly in low- and middle-income countries (LMICs). Approximately 2.9 million cholera cases and 95 000 deaths occur in 69 cholera-endemic countries each year, with a majority occurring in sub-Saharan Africa and South Asia [1]. Cholera transmission is inherently linked to insufficient access to clean water and sanitation services and to unsafe water, sanitation, and hygiene (WASH) practices, and is a significant issue in periurban slums, refugee camps, and places affected by natural disasters [2].

Major infrastructural improvements of WASH in LMICs require significant investment over long periods, and in many resource-limited settings, basic requirements for cholera prevention, such as safe sources of drinking water and sanitation infrastructure, are lacking. In 2017, the Global Task Force on Cholera Control of the World Health Organization launched a global strategy, “Ending Cholera: A Global Roadmap to 2030,” wherein multisectoral approaches for cholera prevention and control, including integrated WASH, oral cholera vaccination (OCV), and surveillance for early disease detection are recommended [3].

Although many clinical studies have demonstrated the protective effectiveness of OCVs, there are far fewer studies examining the empiric impact of feasible WASH improvements. A cluster-randomized trial (CRT) that evaluated in separate arms the effects of OCV, WASH, and combination OCV and WASH in urban Dhaka failed to determine that a WASH intervention added to protection against cholera conferred by OCV alone [4]. However, a previous analysis of a CRT of OCV conducted in an urban slum of Dhaka, Bangladesh, showed that variations in existing WASH practices in the households of a cholera-endemic population were predictive of severe cholera risk [5]. Similarly, household WASH variability in Kolkata, India, where typhoid fever is endemic, was associated with risk of typhoid in household residents [6]. In this analysis we used similar approaches to analyze data generated from a cluster-randomized, placebo-controlled trial of bivalent killed OCV conducted in Kolkata. Here, we reexamine the hypothesis that variations in household WASH already present in urban slums can successfully predict the risk of cholera, which will also be helpful to develop future effective, acceptable, and sustainable WASH interventions in cholera-endemic populations.

## METHODS

### Bivalent Killed OCV Trial in Kolkata, India

A cluster-randomized, double-blind, placebo-controlled trial was carried out in a cholera-endemic area in the urban slums of Kolkata, to assess the safety and efficacy of the bivalent killed OCV (Shanchol, Shanta Biotechnics, India) against cholera. The study area included a population of 107 774 individuals residing in 3933 dwellings in 3 wards (wards 29, 30, and 33) [7]. Each dwelling was considered as a cluster. Residents who were at least 1 year old and not pregnant at the point of assessment were eligible for vaccination. Clusters were randomized to receive 2 doses of either the bivalent killed whole-cell OCV or heat-killed *Escherichia coli* K12 placebo. A 2-dose schedule was administered in 2 rounds, the first from 27 July to 13 August 2006, and the second from 27 August to 10 September 2006. Doses of vaccine and placebo were delivered by oral syringe, without an oral buffer, and stored at 2°C–8°C until administration.

### Demographic Surveillance

A census of the study area was completed before the trial and was updated during interim surveys throughout the study period. Each individual in the study area was assigned a unique study identification number. Baseline demographic information was collected over 2 time points: 2 years prior to the trial in 2003 for residents of wards 29 and 33, and immediately prior to the trial in 2005 for residents of ward 30. Basic demographic information, household-level socioeconomic status, and WASH data from each household were collected at a single time point, during the baseline census or, for households that were not present at the baseline census, during the census update. Household WASH information was not updated during the study. Interim surveys were completed in 2007, 2008, 2009, and 2010–2011, and the surveys recorded birth, death, in-migration, and out-migration events occurring in the study population.

### Disease Surveillance

Surveillance for cholera was carried out in 9 community clinics and in 2 hospitals that provided free diarrheal care services to the population. Private physicians in the area, who provided very little of the care for diarrhea in individuals in the study population, were encouraged to refer patients with diarrhea to the study sites. After enrollment of patients, trained study staff collected relevant clinical details in a systematic fashion. A diarrheal visit was defined as a visit by a patient who reported either ≥3 loose stools, or at least 1 loose stool with blood or 1 loose stool together with evidence of dehydration in the past 24 hours [7]. Diarrheal visits for which the date of onset of symptoms was ≤7 days from the date of discharge for the previous visit were grouped into the same diarrheal episode. Rectal swabs were collected by study physicians and trained staff from all enrolled participants and transported in Cary-Blair media to a laboratory at the National Institute of Cholera and Enteric Diseases (NICED) within 8 hours of specimen collection. At the laboratory, rectal swabs were analyzed for *Vibrio cholerae* by serogroup, biotype, and serotype, by using conventional methods [8, 9].

### Variable Selection to Define “Better” Versus “Not Better” WASH

During the baseline demographic survey, information on 5 polytomous, categorical household variables pertaining to practices and facilities related to WASH were collected. These included source of water for daily use (excluding drinking water), source of drinking water, site of defecation, handwashing practice after defecation, and waste disposal location. We examined each household WASH variable and categorized it into a binary variable termed “better” or “not better” WASH based on substantive judgment of researchers familiar with the study setting and without prior knowledge of cholera incidence rates associated with each variable category. The resulting variable categories were filtered or boiled water for daily use versus other (source of water for daily use); private tap, well, or pump versus other (source of drinking water); private or shared flush toilet versus other (site of defecation); always handwashing with soap after defecation versus other (handwashing practices); and specific place for waste disposal versus other (waste disposal location). All 5 dichotomized WASH variables were then included for consideration in the recursive partitioning model.

### Construction of Decision Tree to Develop Composite WASH Variable in the Training Subpopulation

Households in the control clusters (premises) were randomly divided into “training” and “validation” datasets that were roughly equal in size and mutually exclusive. The number of cholera cases were balanced during selection of the training and validation sets. In the training population, we applied recursive partitioning using the 5 dichotomized household WASH variables to design a decision tree that would predict the risk of cholera among household members during 5 years of follow-up. We imposed a loss function of 1:1 for the cost of false positives to false negatives and required that there were at least 300 observations at each terminal node in the resulting decision tree. To select the optimal decision tree, the minimal complexity parameter was used to prune the model and select the simplest tree with minimum error providing at least 2 terminal nodes in the tree. For the selected rule, we plotted a receiver operating characteristic (ROC) curve and calculated the area under the ROC curve (AUC) to evaluate the ability of the tree to predict cholera in household members. To create a binary composite variable that classified households as having either better or not better WASH based on the cholera risk in inhabitants of those households, a cutoff probability of the incidence of cholera in the terminal nodes of the tree was defined based on maximization of the Youden index for the ROC curve. Subsequently, the algorithm was cross-validated with an estimation of the cross-validation error in 1 of 10 randomly assembled partitions of the training subpopulation. To confirm the reproducibility of sensitivity and specificity of the composite WASH variable developed in the training subpopulation, we tested the variable in a distinct validation subpopulation.

### Protective Association Between WASH and Cholera in the Entire Population

In the entire population residing in control clusters at baseline (closed cohort), we measured the association between residence in a better WASH household and the risk of developing cholera. First, we assessed the similarity of relevant covariates (age [0–4 years, 5–14 years, ≥15 years] at zero time [where zero time is defined as the date of the second dose of vaccine or placebo or median date of second dose of vaccine or placebo in the cluster for nondosed or incomplete dosed individuals], sex, household head occupation, expenditure, household distance to the study clinic) between the training and validation subpopulations to assess population comparability (Supplementary Table 1).

We then measured the association between “better” WASH household status and cholera in the entire control population. To evaluate this association, we analyzed the time from start of follow-up to the first cholera case using the Cox proportional hazard regression model. The model was adjusted for potential confounding covariates, including age, sex, religion, longer than median distance to the clinic, and variables reflecting household socioeconomic status including service holder with stable job, living in own house, and household expenditure higher than median. However, age was included forcedly into the model. We introduced variables into the model by forward stepwise selection, using the 5% significance level for entry in the initial model as well as staying in the final model. Hazard ratios (HRs) for cholera were estimated by exponentiating the coefficient for the composite WASH variable in Cox models and protection was estimated as ([1 − HR] × 100%) with 95% confidence intervals (CIs), and the interval estimates and *P* values were adjusted for the design effect of cluster randomization.

The analysis was performed using the rpart package for decision tree modeling, pROC package for the ROC curve, the survival package for the Cox model, My.stepwise package for variable selection, and dplyr package for data management under R statistical software (version 4.10). For all statistical analyses, a *P* < .05 (2-tailed) was taken as the margin of statistical significance.

### Patient Consent Statement

Written informed consent was obtained from the patient or the guardian of the patient in case of a minor. The study protocol was approved by the ethics committee of NICED, the Health Ministry Screening Committee of India, and the International Vaccine Institute Institutional Review Board, and the trial is registered with ClinicalTrials.gov (NCT00289224).

## RESULTS

### Assembly of Training and Validation Subpopulations

**Figure 1.**
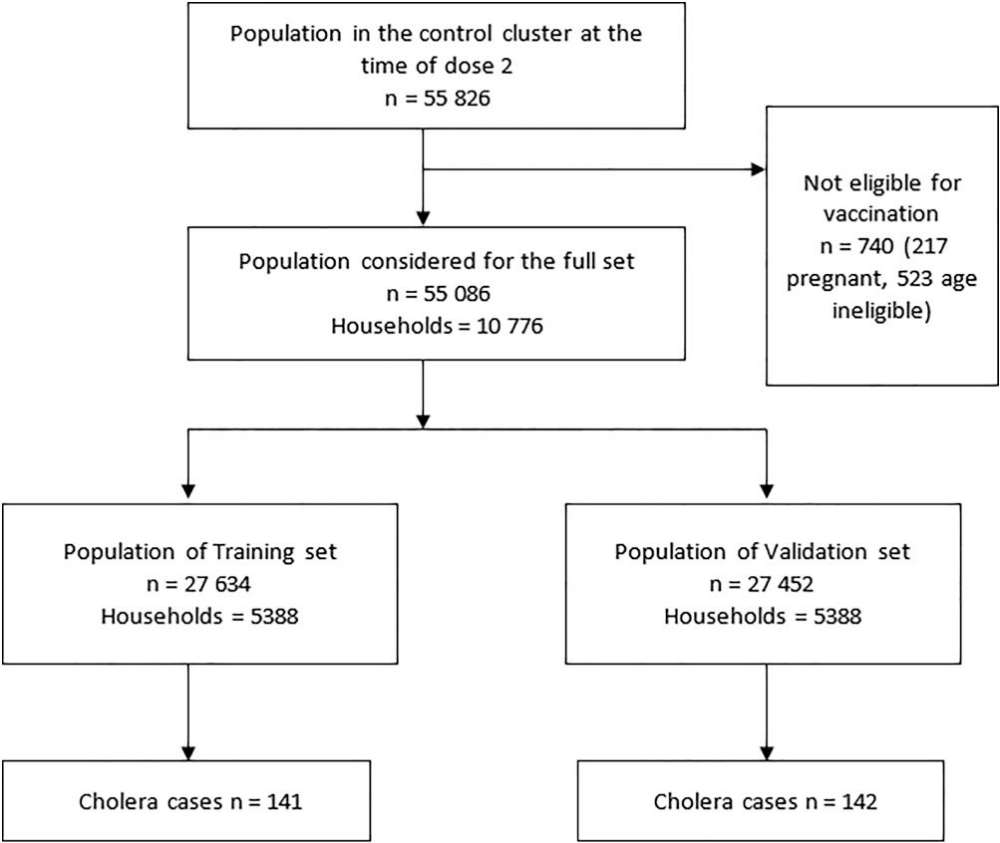
Study population and separation into training and validation population subsets.

During the baseline census, 55 826 individuals from 10 777 households were enumerated from the control clusters. A total of 55 086 residents from 10 776 households were included in this closed cohort analysis (Figure 1). The households were partitioned into training and validation sets at a 50:50 ratio. The training set included 5388 households, 27 634 individuals, and 141 cholera cases and the validation set included 5388 households, 27 452 individuals, and 142 cholera cases. The training and validation subpopulations were comparable and there were no substantive differences in baseline characteristics, including age, sex, religion, and socioeconomic indicators (Supplementary Table 1).

### Model Development for Composite WASH Variable Predicting Cholera

A bivariate analysis was carried out for each dichotomous WASH variable in the training population to measure the association of individual WASH-related variables with the risk of cholera over 5 years of follow-up. Households with a safe source of drinking water, defined as a tap, well, or pump (HR, 0.54 [95% CI, .27–1.08]; *P* = .080), and reporting always handwashing with soap after defecation (HR, 0.71 [95% CI, .47–1.08]; *P* = .113) were indicative of a reduced cholera risk among the individuals residing in those households (Table 1). Other variables—access to a private or shared flush toilet (HR, 0.83 [95% CI, .50–1.39]; *P* = .480), treatment of daily use water by filter or boiling (HR, 0.62 [95% CI, .24–1.56]; *P* = .305), and waste disposal location (HR, 0.75 [95% CI, .32–1.77]; *P* = .517)—were not significantly associated with cholera risk, though the HRs exhibited protective relationships. All 5 variables were included as candidate input variables in the recursive partitioning model to determine a composite variable that was predictive of cholera risk.

**Table 1.** Bivariate Analysis of Water, Sanitation and Hygiene Variables and Cholera Risk in the Training Subset of the Control Population

WASH Variable	Yes				No				HR^a^ (95% CI)	
	No.	Cholera	PY	IR/1000 PY	No.	Cholera	PY	IR/1000 PY	Crude HR^b^	*P* Value
Flush toilet: private/shared	5496	23	16 549	1.4	22 138	118	70 258	1.7	0.83 (.5–1.39)	.480
Drinking water: private tap, well, or pump	4663	13	13 713	0.9	22 971	128	73 095	1.8	0.54 (.27–1.08)	.080
Daily water use: filtered/boiled	3057	9	8691	1.0	24 577	132	78 117	1.7	0.62 (.24–1.56)	.305
Handwashing with soap after defecation: always	19 527	88	60 707	1.4	8107	53	26 101	2.0	0.71 (.47–1.08)	.113
Waste disposal location: specific	26 861	136	84 442	1.6	773	5	2365	2.1	0.75 (.32–1.77)	.517

Abbreviations: CI, confidence interval; HR, hazard ratio; IR, incidence rate; PY, person-years; WASH, water, sanitation, and hygiene.

^a^Estimated from extended Cox proportional hazard model.

^b^Calculated using robust standard error assuming risk of cholera is correlated within clusters.

### Performance of the Composite WASH Variable in Predicting Cholera in the Training Subpopulation

**Figure 2.**
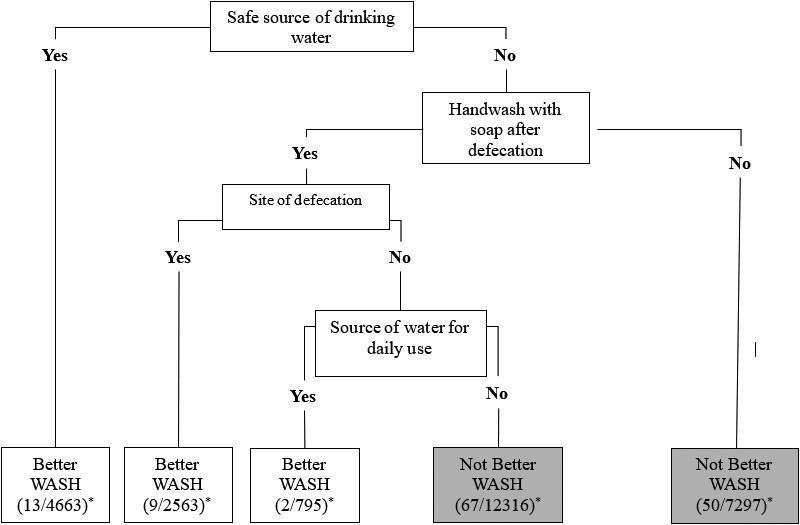
Decision tree for categorization of “better” and “not better” water, sanitation, and hygiene (WASH) using binary WASH variables in the training population. *Number of cholera cases/household population in the training subpopulation.

**Figure 3.**
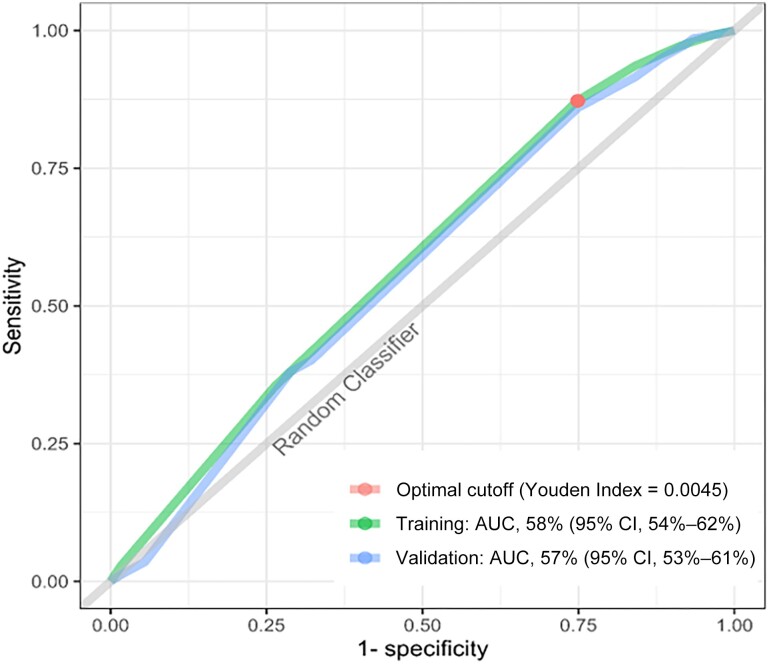
Receiver operating characteristic curve for defining cutoff probability to classify household water, sanitation, and hygiene as a binary variable in the training population. The gray diagonal line represents points with no predictive value. Abbreviations: AUC, area under the receiver operating characteristic curve; CI, confidence interval.

In our dichotomized classification rule, the dominant bifurcation was the for source of drinking water (Figure 2). Individuals residing in households reporting a safe source (tap, well, or pump) of drinking water had a lower risk of cholera irrespective of all other evaluated WASH variables. Additionally, a combination of always handwashing with soap after defecation, site of defecation (private or shared flush), and treatment of water for daily use was also predictive of cholera risk in household members. Site of waste disposal did not impact cholera risk in the model (Figure 2). In the training subpopulation, to select the optimal threshold value for the predictive rule, we used the Youden index for maximization of sensitivity and specificity on the ROC curve, which corresponded to a cutoff probability of cholera incidence of 0.0045. The AUC was 58% (95% CI, 54%–62%) for the training set (Figure 3). Sensitivity (the proportion of participants developing cholera who lived in households with not better WASH) of the rule in the training population was 83% (95% CI, 76%–89%) and specificity (the proportion of participants not developing cholera who lived in households with better WASH) was 29% (95% CI, 29%–30%). In the validation population, sensitivity and specificity were similar at 84% (95% CI, 77%–89%) and 29% (95% CI, 28%–29%), respectively.

### Prediction of Cholera Incidence by Household WASH Status in the Entire Population

Analysis of the entire population of control clusters showed that the overall incidence of cholera in residents of better WASH households was 1.0 cholera cases per 1000 person-years, compared to 1.9 cholera cases per 1000 person-years in residents of not better WASH households. The crude HR of culture-confirmed cholera by residence in a better WASH household was 0.51 (95% CI, .35–.74; *P* < .001). The HR adjusted for potentially confounding variables was 0.70 (95% CI, .49–.99; *P* = .048). After stratifying by age at baseline, we found that residence in a better WASH household had the largest impact on cholera risk in young children and that this effect progressively declined with age. The adjusted HR was 0.32 (95% CI, .14–.74, *P* = .007) in the age group 0–4 years, 0.55 (95% CI, .25–1.22; *P* = .143) in the age group 5–14 years, and 0.98 (95% CI, .62–1.53; *P* = .919) in the age group ≥15 years (Table 2). A significant negative trend (1-tailed *P* = .035) was found by linear regression of age group and effect of better WASH on cholera risk.

**Table 2.** Household-Level Protection Against Cholera Associated With “Better” Water, Sanitation, and Hygiene in the Control Population

Age Group, y	Better WASH				Not Better WASH				HR (95% CI)			
	No.	Cholera	PY	IR/1000 PY	No.	Cholera	PY	IR/1000 PY	Crude HR	*P* Value	Adjusted HR^a^	*P* Value
All	15 832	47	48 230	1.0	39 254	236	123 803	1.9	0.51 (.35–.74)	<.001	0.70 (.49–.99)^a^	.048
0–4	827	6	2810	2.1	2421	61	8245	7.4	0.29 (.13–.67)	.003	0.32 (.14–.74)^b^	.007
5–14	2297	7	8178	0.9	7859	61	28 848	2.1	0.41 (.19–.88)	.023	0.55 (.25–1.22)^c^	.143
≥15	12 708	34	37 242	0.9	28 974	114	86 711	1.3	0.69 (.44–1.08)	.102	0.98 (.62–1.53)^d^	.919

*P* values are adjusted for the design effect of cluster randomization.

Abbreviations: CI, confidence interval; HR, hazard ratio; IR, incidence rate; PY, person-years; WASH, water, sanitation, and hygiene.

^**a**^Adjusted by age group, service holder has a stable job, household expenditure higher than median, longer than median distance to the clinic.

^b^Adjusted by household expenditure higher than median.

^c^Adjusted by service holder has a stable job, household expenditure higher than median, longer than median distance to the clinic.

^d^Adjusted by religion, household expenditure higher than median, longer than median distance to the clinic.

## DISCUSSION

Using existing variability in household WASH properties ascertained at baseline, we developed a dichotomized classification rule that predicted the 5-year risk of cholera in household residents of the slum population under study. Household source of drinking water (defined as a tap, well, or pump) was the strongest determinant of cholera risk in household members, although even in the absence of improved drinking water, we demonstrated that consideration of other WASH characteristics with household source of drinking water added to the predictive strength of the decision rule. Residence in a household with better WASH was associated with 30% (95% CI, .49–.99; *P* = .048) (Table 2) lower risk of cholera compared to residence in a not better WASH household over a 5-year period, and risk reduction was inversely related to age at zero time.

These findings suggest that affordable and sustainable improvements in household WASH, as they are already existing within the population, can have a major impact on cholera risk. This suggests that certain WASH properties may have an outsized role in disrupting cholera transmission and that, even in absence of an important WASH determinant such as safe source of drinking water, better WASH can be achieved with improvement of other practices (safe source of water for daily water use, flush toilet use, and handwashing with soap after defecation).

Young children aged 0–4 years, who were at highest risk for cholera, experienced the greatest benefit of improved household WASH characteristics. The highest incidence of cholera was in this age group, and eventually the greatest number of cholera cases was averted by better WASH, with the highest risk reduction in children 0–4 years of age. Both risk reduction associated with better household WASH and the underlying incidence of cholera decreased in older age groups. The reduced protective association of household WASH with risk of cholera by age may be due to the increased mobility of older persons outside of the reach of household WASH behaviors and practices.

Improved household WASH predicted 5-year cholera risk reduction in all age groups. The extended impact of these WASH characteristics, which were ascertained at baseline, suggests that naturally achieved WASH improvements are sustainable and can have prolonged effects.

The definition of better WASH in this study is different from the definition derived in a similar study of cholera conducted in Dhaka, Bangladesh [5]. These differences may be related to variations and differences in socioeconomic and cultural characteristics, the study period, and differences in WASH variables collected at baseline. However, in an earlier study of typhoid fever in the same population in Kolkata, we found that the definition of better WASH that was associated with a reduced risk of typhoid fever in household members was similar [6]. This finding supports the notion that minor improvements in household WASH are effective in preventing multiple disease targets.

There are a limited number of high-quality studies evaluating the impact of WASH interventions on cholera. A majority of studies describe water quality interventions without addressing other routes of transmission, and water treatment interventions were limited by low effectiveness and poor adherence [10]. On the other hand, the protective effect of handwashing with soap has been demonstrated in several studies, highlighting a simple and relatively affordable opportunity to prevent person-to-person transmission as well as food and household water contamination [10]. Existing guidelines on cholera control are focused primarily on preventing environmental contamination by cases and minimizing outbreak potential, and consistent recommendations to prevent household and community transmission were limited [11]. Our analysis was noninterventional but assessed multiple naturally occurring potentially relevant behaviors and facilities interacting to reduce the risk of cholera and thereby provides public health policymakers with guidance in designing multifactorial WASH interventions based on already existing salutary practices.

There are several important limitations to consider when interpreting our findings. First, the household WASH variables were collected in the trial only as covariates for assessment of OCV protection against cholera in a cluster-randomized vaccine trial. These variables may be simplistic representations of more complex WASH behaviors and may be unable to capture the full extent of WASH variations between households and therefore make our findings conservative. Second, the household WASH variables were obtained at baseline and were not updated throughout the study period. Moreover, the methods used to collect WASH information may have been subject to response bias. We assume that systematic secular improvements in WASH over time or misclassification of households due to bias in data collection, or both, would overestimate the proportion of better WASH households, thereby making our findings conservative. Third, we measured naturally occurring variations in the level of household WASH; therefore our findings may have been confounded by other elements, such as healthcare-seeking behavior, that we were not able to control for in the analyses. However, our findings were not explained by potential confounding variables that reflected socioeconomic status of the population. Fourth, the original WASH variables were polytomous and they were dichotomized for the purposes of this analysis; dichotomization may have led to loss of information, potentially making our analyses conservative. Last, it is possible that households with higher socioeconomic status and, therefore better WASH characteristics, were more likely to seek care at private facilities outside of the study network. Systematically missing cases from this population subset would result in an overestimation of the protective effect of household WASH. However, in the study area, 9 clinics and 2 hospitals provided cost-free diarrheal care and the population was highly sensitized to the availability of high-quality care for diarrheal disease from these health centers. Additionally, private practitioners were frequently engaged and encouraged to refer diarrheal patients to 1 of the study centers. Therefore, the number of missed cholera cases due to healthcare-seeking behavior at alternative centers is likely to be low.

Despite these limitations, our analyses have several strengths. First, we used the data from a prospective, cluster-randomized controlled trial of OCV that was carried out in a well-defined population where the data were collected systematically in a comprehensive manner. Second, the population in the study area was relatively stable over the 5 years of follow-up, suggesting that an effect of migration was unlikely to have affected our analysis. Third, the external validation of the WASH prediction rule using a separate validation set showed a similar level of sensitivity and specificity, which indicates that the prediction rule was not overfitted to the training population dataset.

Our findings indicate that the existing and therefore achievable WASH improvements are associated with a sustained reduced risk of cholera in a cholera-endemic setting. The methodology used in our study has been replicated in several populations and for evaluation of multiple disease targets. Whether or not these WASH enhancements affect the protection against cholera afforded by OCV is an essential question to evaluate going forward. Evidence for protection against cholera by incremental and inexpensive improvements of household-level WASH should be investigated in future study designs with WASH interventions.

## Supplementary Material

ofad535_Supplementary_Data

## Supplementary Data

Supplementary materials are available at *Open Forum Infectious Diseases* online. Consisting of data provided by the authors to benefit the reader, the posted materials are not copyedited and are the sole responsibility of the authors, so questions or comments should be addressed to the corresponding author.

## Notes

***Acknowledgments.*** The International Centre for Diarrhoeal Disease Research, Bangladesh (icddr,b) is grateful to the governments of Bangladesh and Canada for providing unrestricted support.

***Disclaimer.*** The funders had no role in study design, data collection and analysis, decision to publish, or preparation of the manuscript.

***Financial support.*** This study was funded by the Bill & Melinda Gates Foundation (grant number INV 025388).
