# Supplementary material for: Better Existing Water, Sanitation, and Hygiene Can Reduce the Risk of Cholera in an Endemic Setting: Results From a Prospective Cohort Study From Kolkata, India
Source: Open Forum Infect Dis. 2023 Nov 21;10(11):ofad535. doi: 10.1093/ofid/ofad535 (PMC10662546; doi:10.1093/ofid/ofad535)
Supplement: ofad535_Supplementary_Data [file ofad535_supplementary_data.docx]

**Supplement Table 1.** Baseline characteristics of the training, and validation subpopulations in the control population

|  | Training subpopulation | Validation subpopulation |
| --- | --- | --- |
| Characteristics | Overall, N = 27,634 | Overall, N = 27,452 |
| **Age group** |  |  |
| 0-4,yrs | 1,568 (5.7%) | 1,680 (6.1%) |
| 5-14,yrs | 5,095 (18%) | 5,061 (18%) |
| 15+,yrs | 20,971 (76%) | 20,711 (75%) |
| **Gender** |  |  |
| Female | 12,737 (46%) | 12,731 (46%) |
| Male | 14,897 (54%) | 14,721 (54%) |
| **Religion** |  |  |
| Others | 10,034 (36%) | 10,047 (37%) |
| Hindu | 17,600 (64%) | 17,405 (63%) |
| **Living in own house** |  |  |
| No | 18,098 (65%) | 18,315 (67%) |
| Yes | 9,536 (35%) | 9,137 (33%) |
| **Service holder have a stable job** |  |  |
| No | 15,350 (56%) | 15,456 (56%) |
| Yes | 12,284 (44%) | 11,996 (44%) |
| **HH* expenditure higher than median** |  |  |
| No | 15,051 (54%) | 15,414 (56%) |
| Yes | 12,583 (46%) | 12,038 (44%) |
| **Longer than median distance to the clinic** |  |  |
| No | 13,723 (50%) | 13,801 (50%) |
| Yes | 13,911 (50%) | 13,651 (50%) |

*HH- House Hold
